# Identification of HPV-16 in Borderline Mucinous Cystic Neoplasm of Pancreas

**Published:** 2007-03

**Authors:** Tommy R. Tong, Anthony Chan, Tak-wing Lai, Olivia W. Chan, Kam-cheong Lee, Stephen TH Lo, Rosanna Lung, Jackson YW Li, Tat-chong Chow

**Affiliations:** 1 *Departments of Pathology, Princess Margaret Hospital, Hong Kong, China;*; 2 *Department of Pathology, Prince of Wales Hospital, Hong Kong, China;*; 3 *Departments of Surgery, Princess Margaret Hospital, Hong Kong, China;*; 4 *Department of Pathology, Nethersole Hospital, Hong Kong, China*

**Keywords:** pancreatic mucinous cystic neoplasm, human papillomavirus, pancreas, carcinoma

## Abstract

Pancreatic mucinous cystic neoplasms (PMCN) predominantly affect women in the reproductive age, are located in the body and tail of the pancreas, and share morphological features with similar tumors of the ovary. We report the detection of human papillomavirus (HPV) using several different PCR protocols in a borderline PMCN from a female patient. Type-specific PCR demonstrated the HPV to be type 16. If confirmed by others, this group of neoplasms might become preventable by HPV vaccination.

## INTRODUCTION

Pancreatic cancers are a heterogeneous group of tumors with significantly different clinical features, anatomic locations, histopathological features, molecular pathogenesis, and prognosis. The majority of these tumors are highly malignant adenocarcinomas, occur in the head of the pancreas (PANCA), and have a higher incidence in men. Pancreatic mucinous cystic neoplasms (PMCN) and solid-pseudopapillary tumor are rare tumors which occur mostly in women, the latter nearly exclusively so. Early detection and complete resection of these tumors offer a much better clinical outcome than PANCA. These differences suggest that different etiologic agents and intracellular pathways may be involved in tumorigenesis.

Recently, a series of ovarian adenocarcinomas was reported in which oncogenic HPV were found. Synchronous and metachronous endocervical adenocarcinomas positive for the same HPV types were identified (1). We posit HPV as an etiologic agent in a recent case of PMCN, which histologically resembles ovarian mucinous adenocarcinomas.

## CLINICAL AND PATHOLOGIC FINDINGS

**Figure 1:**
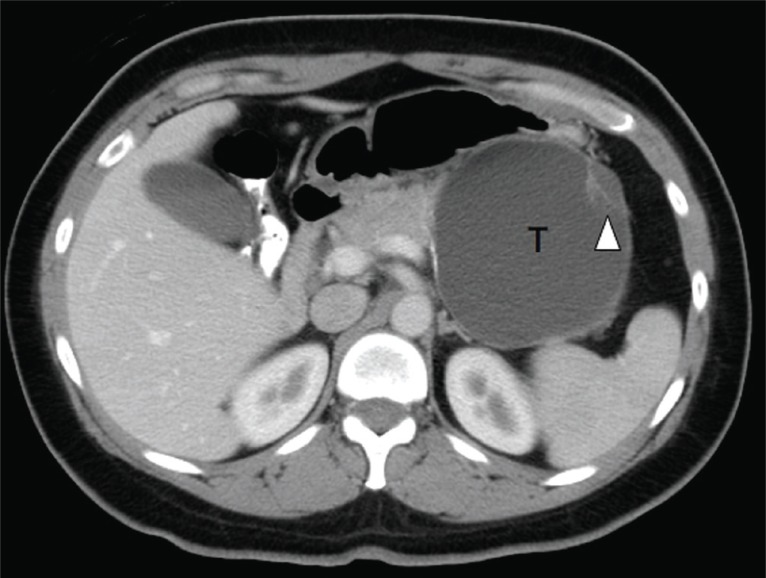
Computed tomography of the abdomen. This image shows a large cystic tumor in the left upper quadrant of the abdomen (indicated by T, right side of image). Arrowhead points to daughter locules.

**Figure 2:**
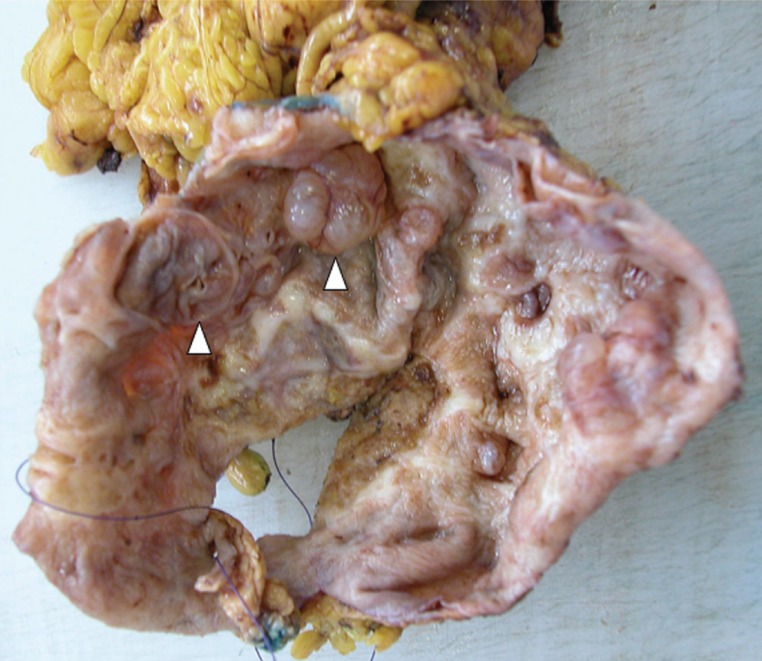
Macroscopic appearance of borderline pancreatic mucinous cystic neoplasm. Note daughter cysts within opened cyst. Arrowheads points to daughter locules.

**Figure 3:**
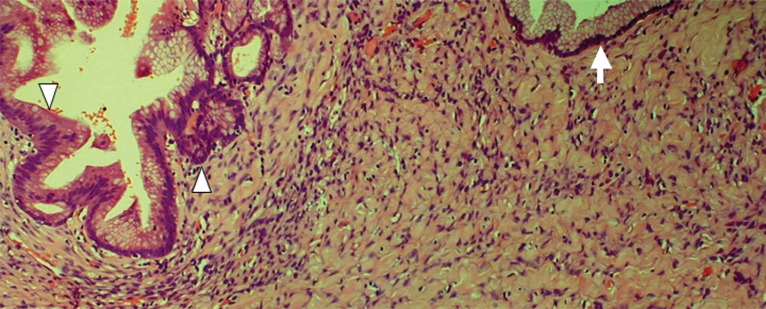
Microscopic appearance of tumor. A representative field depicts benign mucinous lining (arrow, top right) and stroma with accentuation of cellularity immediately subjacent to the mucinous lining. Borderline areas (arrowheads) with nuclear stratification and moderate atypia are shown on the left. Non-neoplastic pancreas is seen in the bottom (hematoxylin-eosin, original magnification ×100).

The patient is a previously healthy 45 year-old woman who experienced epigastric pain for several days before presentation. Physical examination showed a firm left upper quadrant abdominal mass. There was no tenderness or ascites. No lymphadenopathy was found. Rectal and vaginal examinations were negative for pelvic mass. Serum amylase level was normal. Abdominal ultrasonography revealed a large cystic lesion at the tail of the pancreas. Computerized axial tomography confirmed the presence of a thin walled cystic lesion with peripheral septations arising from the pancreatic tail, measuring 12 × 11 × 9 cm (Fig. 1). Distal pancreatectomy was performed. Grossly, the tumor was cystic, comprising one large locule and several smaller daughter cysts (Fig. 2). The contents were mucoid. The resection margins were not involved. Microscopically, the epithelial lining was composed of a single layer of columnar epithelial cells with foveolar architecture and focal intestinal differentiation (Fig. 3). The stroma immediately underlying the epithelial lining was focally cellular and resembled ovarian stroma. The patient recovered from the operation uneventfully. Four weeks after surgery, she consented to a Papanicolaou smear and underwent colposcopic examination of the cervix, both of which were normal. She remained disease free for at least six months.

## MOLECULAR PATHOLOGICAL FINDINGS

Tissue blocks with tumor and non-neoplastic pancreatic tissue were selected for HPV detection by general primer and type-specific PCR as previously reported (2-4). In brief, ten 10 m paraffin sections were made from the paraffin blocks and DNA extracted after the standard proteinase K-phenol chloroform treatment. The purified DNA was precipitated with absolute ethanol and dissolved in 100 μl distilled water. 10 μl of the extracted DNA was used for the detection of HPV by PCR method. The general primer GP5+/6+, which amplifies a region of 140 to 150 bp in the L1 open reading frame of a broad spectrum of HPV genotypes were employed initially (5). For GP5+/6+ negative reactions, the second general primer PCR SPF1/2, which has a higher sensitivity and amplifies a 65 bp segment of the L1 region was performed (6). In addition, type-specific PCR was employed for positive specimens (7). The type-specific PCR consisted of two separate reactions, which detect HPV 6, 16 and 33, and HPV 11, 18 and 31, respectively. To assess the quality of extracted DNA, human beta-actin PCR was performed in parallel. To prevent carryover contamination, tissue blocks were cut with new blades after prior decontamination of the microtome and instruments. DNA extraction and purification, master-mix set-up, PCR amplification, as well as post-amplification gel electrophoresis were all carried out in dedicated areas. Uni-directional workflow for the PCR processes was followed. All pipetting works were conducted with the use of disposable aerosol-resistant micropipette tips.

**Figure 4:**
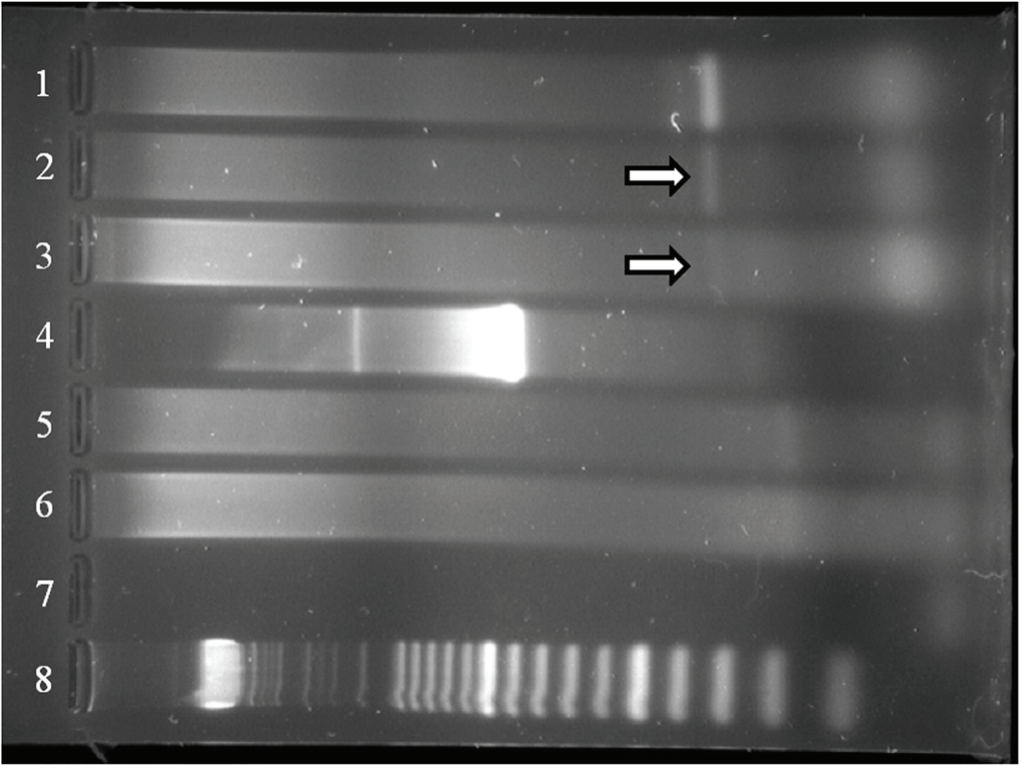
Amplification of HPV targets as demonstrated by gel electrophoresis following PCR with GP5+/6+ and MY09/11. Lanes 2 and 3 show weak and faint bands (arrows), respectively, of tumor tissue amplified with GP5+/6+ primer pairs. The same tissue was negative using MY09/11 primer pairs (lanes 5 and 6). The MY09/11 primers are annealed further apart in the HPV genome, which may be extensively cross-linked in formalinfixed paraffin-embedded material, preventing amplification by PCR using these primers. Lanes 1 and 4 show location of bands for positive control using GP5+/6+ and MY09/11 primer pairs. Lane 7 is negative control. Lane 8 represents DNA size ladder.

**Figure 5:**
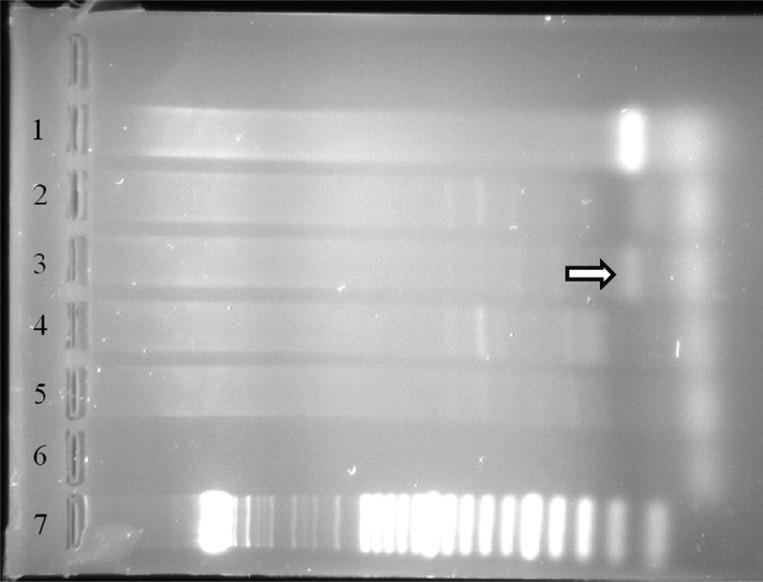
Amplification of HPV targets as demonstrated by gel electrophoresis following PCR with SPF1/2. Lane 3 shows position of band from tumor tissue (arrow). Lane 2 is a duplicate run, which is negative. Lanes 4 and 5 are negative results from a different tumor block. The inconstant results suggest that HPV may be present in low copy numbers. Lane 1 depicts the location of the band for the positive control. Lane 6 is negative control. Lane 7 represents DNA size ladder.

**Figure 6:**
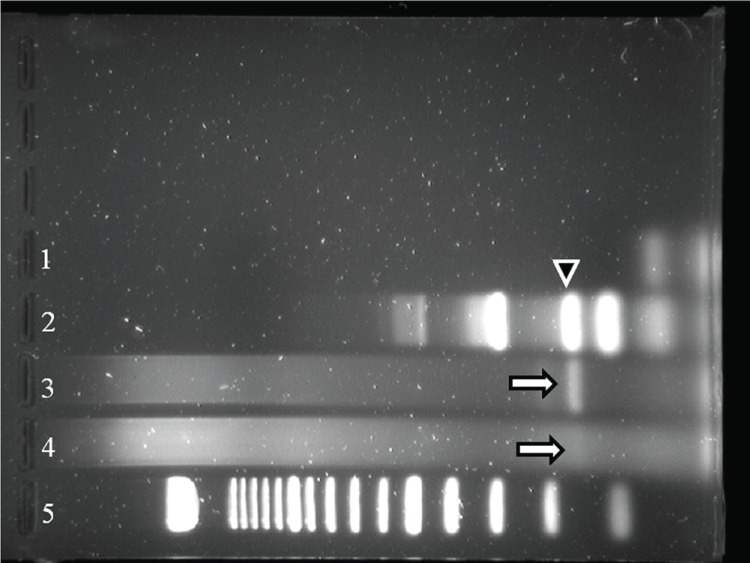
Type-specific PCR identifying HPV 16 in PMCN. Lanes 3 and 4 show strong and faint bands (arrows) from undiluted and diluted starting material from tumor, respectively. Lane 1, negative control. Lane 2, positive control, with the position of HPV 16 indicated with arrowhead. Lane 5 represents DNA size ladder.

HPV was identified in tumor blocks by general primer PCR reactions employing GP5+/6+ (Fig. 4) and SPF1/2 primers (Fig. 5). Type-specific PCR identified the HPV as type 16, a member of the high-risk HPVs (Fig. 6). Non-neoplastic pancreatic tissue and water negative controls were consistently negative for HPV. General primer PCR employing primer pairs MY09/11 was negative (Fig. 4).

## DISCUSSION

Pancreatic mucinous cystic neoplasms are of unknown etiology and are much more common in women than in men. Its location in the body and tail of the pancreas is in contrast to conventional pancreatic adenocarcinoma of the head of the pancreas (PANCA), which has a higher incidence in men (8). The evolution of PMCN is slow, giving a larger window for curative resection. Malignant progression takes place in the epithelial component but can also occur in the mesenchymal component, giving rise to sarcomas such as malignant fibrous histiocytoma. Fully malignant tumors are associated with oncogenic K-ras mutations and over-expression of EGFR and inactivation of tumor suppressors p53 and DPC4.

In terms of molecular pathways involved, PMCN differs from PANCA in being mostly negative for immunohistochemical expression of p53 (10). In uterine cervical carcinomas, which almost exclusively contain oncogenic HPV, high-risk HPV E6 targets p53 for proteasomal degradation, keeping p53 at a low level. As expected, most pre-malignant cervical lesions and a significant fraction of invasive cervical carcinomas are negative for p53 overexpression (11). This lends support to a possible etiologic link between HPV and PMCN. However, it is also possible that the p53 pathway may be alternatively disrupted, for example by over-expression of hDM2.

Recently, a series of ten ovarian adenocarcinomas was reported, having synchronous and metachronous HPV-induced endocervical adenocarcinomas, some masquerading as pre-invasive lesions (1). Pancreatic cancers, including an example of PMCN have occurred synchronously or metachronously with ovarian mucinous adenocarcinomas (12). There was no mention of endocervical adenocarcinoma in the report, which attributes the ovarian cancers to metastasis from the pancreatic cancers. In another report, PMCN occurred synchronously with ovarian mucinous cystadenoma, thought to represent independent primaries (13). Thus, ovarian carcinomas have occurred synchronously with endocervical adenocarcinoma and with PMCN.

The hypothesis of PMCN being caused by HPV is novel, as we were not able to find similar case report in the English language medical literature. How the pancreas is infected by HPV is unknown. It is more likely to be the seat of metastasis from an occult primary elsewhere that is caused by HPV. A search for occult tumors, for example in the endocervix, and testing for HPV in PMCN will help to confirm or refute an etiologic link of oncogenic HPV and PMCN. On June 8, 2006, the U.S. Food and Drug Administration licenses the recombinant HPV vaccine Gardasil® as a vaccine for the prevention of cervical cancer and other diseases in females caused by HPV. The vaccine was developed against HPV types 6, 11, 16 and 18. Clinical trials have demonstrated near 100% efficacy in protection against precancerous lesions caused by types 16 and 18, which are oncogenic. If PMCN is indeed caused by high-risk HPVs, vaccination against these epitheliotropic viruses might also prevent these tumors.

In summary, employing different PCR protocols, we consistently detected human papillomavirus, typed as the oncogenic HPV16, in a pancreatic mucinous cystic neoplasm. Further studies of these tumors are warranted.
